# Usability and feasibility of the Test of Adherence to Inhalers (TAI) Toolkit in daily clinical practice: The BANANA study

**DOI:** 10.1038/s41533-024-00372-z

**Published:** 2024-05-28

**Authors:** Maria Achterbosch, Susanne J. van de Hei, Boudewijn J. H. Dierick, Janwillem W. H. Kocks, Maarten van den Berge, Huib A. M. Kerstjens, Sandra Been-Buck, Titia Klemmeier, Liset van Dijk, Job F. M. van Boven

**Affiliations:** 1 Department of Clinical Pharmacy & Pharmacology, University of Groningen, University Medical Center Groningen, Groningen, The Netherlands; 2 Groningen Research Institute for Asthma and COPD (GRIAC), Groningen, The Netherlands; 3 Medication Adherence Expertise Center of the Northern Netherlands (MAECON), Groningen, The Netherlands; 4 General Practitioners Research Institute, Groningen, The Netherlands; 5 Observational and Pragmatic Research Institute, Singapore, Singapore; 6 Department of Pulmonary Diseases & Tuberculosis, University of Groningen, University Medical Center Groningen, Groningen, The Netherlands; 7 Department of Pulmonary Diseases, Martini Hospital, Groningen, The Netherlands; 8 Nivel, Utrecht, The Netherlands; 9 Department of PharmacoTherapy, -Epidemiology & -Economics (PTEE), Faculty of Science and Engineering, Groningen Research Institute of Pharmacy, University of Groningen, Groningen, the Netherlands

**Keywords:** Patient education, Asthma

## Abstract

The Test of Adherence to Inhalers (TAI) Toolkit links an adherence measurement instrument (the TAI) to proven effective interventions for different causes of non-adherence to inhaled medication. This study aimed to assess the usability and feasibility of the TAI Toolkit in clinical practice. The TAI Toolkit was piloted in eight primary and secondary care settings. Each study site included 10 patients with asthma and/or COPD and suspected non-adherence. Healthcare professionals (HCPs) recorded clinical data and TAI Toolkit outcomes. Data on usability and feasibility were collected in semi-structured interviews and with the System Usability Score (SUS). Of the included patients, 81% were non-adherent, and sporadic non-adherence was the most common (69%). The TAI Toolkit was valued with a mean SUS-score of 85.9 by the HCPs. They found the toolkit to ‘be visually attractive’, ‘easy-to-use’ and ‘give insight into patients’ adherence’, thereby offering good potential for its use in clinical practice.

## Introduction

Inhaled medication is the cornerstone of the treatment of patients with asthma and COPD, yet adherence is often suboptimal^1^. Importantly, non-adherence greatly impacts both patients and society. Amongst others, suboptimal adherence has been associated with poor symptom control, more frequent exacerbations, higher mortality and increased costs^2,3^.

For healthcare professionals, management of non-adherence can be challenging given that it has a great variety of causes, including socioeconomic, therapy-related and health system-related factors^4^. To identify non-adherence and the type of non-adherence, the Test of Adherence to Inhalers (TAI) has been developed^5^. This questionnaire is the only adherence questionnaire specifically designed for inhalation medication. Although the TAI identifies non-adherence and the type of non-adherence, it does not provide solutions. Therefore, we developed the TAI Toolkit^6^. The toolkit is based on an extensive literature review of successful adherence-enhancing interventions evaluated in randomised controlled trials. The use of the toolkit is to support healthcare professionals in identifying and managing non-adherence by practical, effective and efficient tailoring of the right intervention to each patient’s adherence need^6^. This “TAI Toolkit” contains a variety of interventions for the different types of non-adherence, but has not been tested and implemented into daily practice.

The primary aim of this study was to identify the usability and feasibility of the TAI Toolkit by healthcare professionals in primary and secondary care settings and uncover what is needed to optimise the toolkit for daily use. Our secondary aims were: (i) to gain more insight into the type of patients the TAI Toolkit could be beneficial for, (ii) to gain more insight into the types of non-adherence that are most prominent and (iii) to explore which interventions are mostly advised.

## Methods

### Study design and setting

In this observational usability study, the TAI Toolkit was piloted in eight primary and secondary care organisations across the Netherlands. In each organisation, one or two healthcare professionals (HCPs)—e.g. physicians, nurses, specialised nurses or physician assistants—tested the TAI Toolkit during their routine clinical activities in ten consecutive eligible patients. Study sites were recruited via the authors’ networks.

### Patient inclusion and exclusion criteria

To mimic the real-world as much as possible, inclusion criteria for patients were held to a minimum. The following inclusion criteria were applied: ≥18 years, having a physician diagnosis of asthma and/or COPD, using inhaled maintenance medication (LABA, LAMA and/or ICS) for at least 3 months and having a healthcare provider suspicion of non-adherence (e.g. high rescue medication use, uncontrolled disease, exacerbations, prescription/dispensing review). No explicit exclusion criteria were applied.

### Development of the TAI Toolkit

**Fig. 1:**
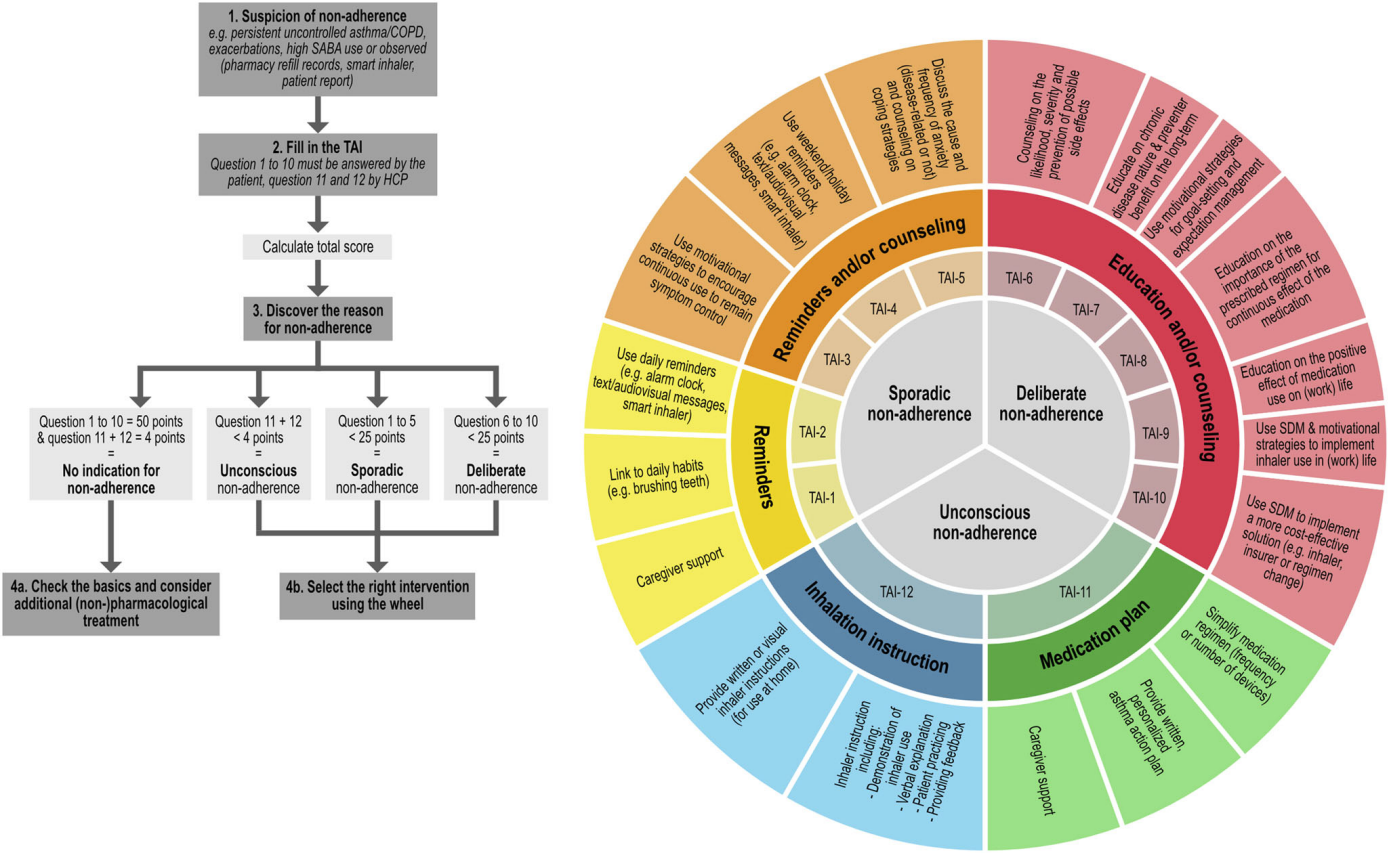
Test of Adherence (TAI) intervention wheel (reproduced without any changes from Van de Hei et al. [6]).

With the validated TAI questionnaire, it is possible to identify non-adherence in patients with COPD and asthma (a TAI-10 score <50 or TAI-12 score <54) and to determine why the patient is non-adherent (score of lower than 5 on one of the sub-items), but it does not give HCPs any guidance on how to support and guide the individual patient with more adherent behaviour^5^. Previously, Van de Hei et al. have reviewed effective interventions developed to enhance medication adherence in patients with asthma and/or COPD and are tested in randomised controlled trials. Seven types of interventions were identified: (1) motivational strategies, (2) shared decision-making, (3) simplifying medication regimen, (4) feedback on medication use, (5) reminders, (6) education, and (7) multiple component interventions^6^. Identified interventions were linked with the 12 TAI items and visually summarised (Fig. 1). This resulted in the first prototype of the TAI Toolkit. The prototype has been evaluated by a panel of eight HCPs (physicians, nurses and pharmacists) and scored using the System Usability Scale (SUS). A median score of 71.4 (range 57.5–80.0) was found, meaning the TAI Toolkit was deemed to have good theoretical usability^6^.

**Fig. 2:**
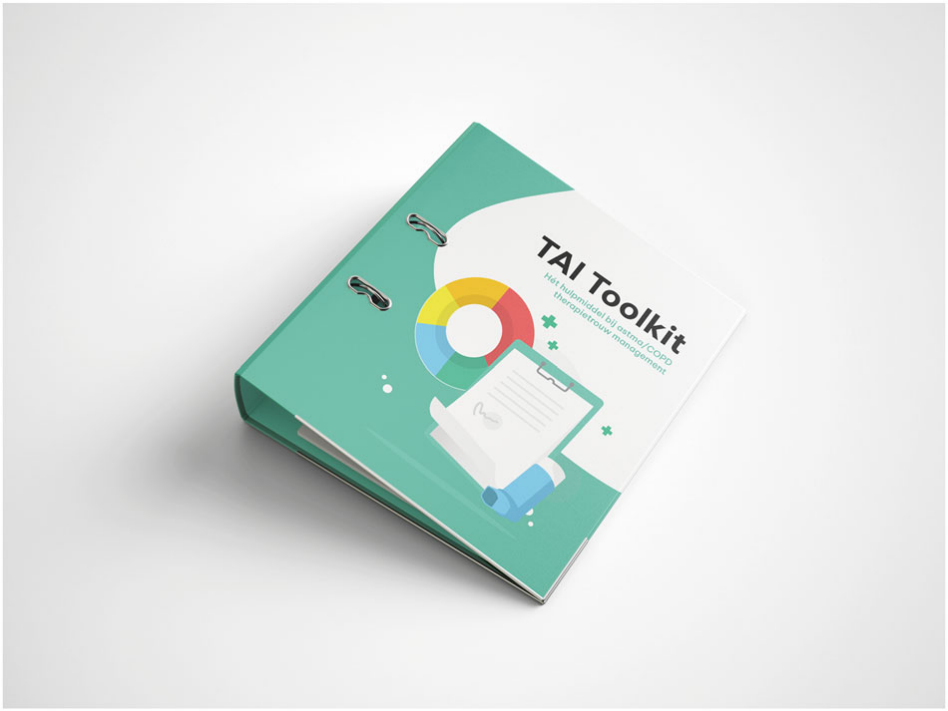
TAI Toolkit prototype.

In the current study, we further developed the TAI Toolkit into an actual practical physical toolkit to be used in daily clinical practice. The TAI Toolkit was translated into Dutch and extended with more information and guidance concerning the use of the Toolkit, background on the individual TAI-items and practical tips and tricks to provide the interventions. The TAI Toolkit was designed as a binder with removable tabs and, therefore easily accessible and adjustable to use as “desktop helper” (Fig. 2). The binder starts with the TAI questionnaire followed by tabs with the following content in order of appearance; (1) introduction text, (2) user instruction, (3) intervention wheel (Fig. 1), (4) background information on the different types of non-adherence (sporadic, deliberate and unconscious), (5) an overview of the TAI-items and matching interventions, and (6) the interventions with explanation of the intervention, helpful tools and where to find those tools and points of attention when executing the intervention.

### TAI Toolkit training

The participating HCPs received online training on the use of the TAI Toolkit. This 1-h training consisted of an introduction to the use of the TAI and the TAI Toolkit in addition to explaining the study procedures. Given one of the goals of this study was to identify the unmet educational needs to use the TAI Toolkit in routine daily practice, no training on the TAI Toolkit recommended interventions (e.g. shared decision making, inhalation instruction, information and instruction) was provided. The need for additional training was evaluated during end-of-study interviews.

### Study procedures

After completing the training, HCPs tested the TAI Toolkit during regular consultations until ten consecutive eligible patients were included per centre. Testing the TAI Toolkit implied using the TAI questionnaire, then applying the TAI Toolkit and selecting an intervention to improve adherence. Of note, the execution of the selected intervention was not part of this study.

In addition, demographic and clinical patient data were collected. After data-collection, HCPs were individually online interviewed using a semi-structured interview. The interview was structured according to the RE-AIM framework with each section including related questions concerning the usability and feasibility of the TAI Toolkit.

### Outcomes

Outcomes focused on (i) patient characteristics, (ii) feasibility and implementation characteristics and (iii) medication adherence items identified and interventions required.

Patient characteristics concerned demographic data, age and gender. Clinical data collected included the indication for inhalation medication, comorbidity data, number and type of medications, last known spirometry, symptom control (Asthma Control Questionnaire [ACQ]/Clinical COPD Questionnaire [CCQ])^2,5^ and exacerbations data. Socioeconomic data included educational level, household composition and social support (Appendix 1).

To evaluate the implementation feasibility of the TAI toolkit, the *reach, effectiveness, acceptability, implementation* and *maintenance* (RE-AIM) framework was used^7,8^. For each component—except effectiveness—two or more questions were phrased to ask the HCPs during the end-of-study interview. The effectiveness of the TAI Toolkit was not measured since this was not the goal and was also beyond the scope of the current study. However, for each intervention suggested in the TAI Toolkit, a positive effect on medication adherence has been found previously^6^.

For *reach*, HCPs were asked to give an estimation of how many patients with asthma and COPD they see on a weekly base and how many of those patients they suspected to be non-adherent.

For *acceptability*, HCPs were asked to score the TAI Toolkit with the System Usability Scale (SUS). The SUS is a 10-item 5-point Likert-scale with 100 being the maximum score and scores >68 deemed as “good” usability. After coding all items in the same direction, lower scores mean less perceived effectiveness, efficiency or satisfaction and higher scores mean well-perceived effectiveness, efficiency or satisfaction with the product^9^. Additionally, HCPs were asked to mention three positive and three points of improvement for the TAI Toolkit. For the domain *implementation*, open and multiple options questions concerned the design of the toolkit, when to use it, who is best suited to use the toolkit, whether training is necessary and whether the toolkit and its interventions were operable. For *maintenance*, two questions were composed; one on how the TAI Toolkit could best be integrated within healthcare and the specific setting and one on how to deal with future adjustments of the TAI Toolkit (Appendix 2).

For each patient, HCPs were asked to state the reason why they suspected medication non-adherence.

HCPs registered responses on the TAI and its total score. From this, the types of non-adherence (sporadic, deliberate and unconscious) were identified. The relation between the different non-adherence subtypes was assessed with the Fischer exact test. Additionally, HCPs registered the three TAI items that most frequently scored the lowest and which related intervention(s) they selected with the TAI Toolkit. Both medication adherence and the outcome of the TAI Toolkit were assessed for the total population and for the subgroups of asthma and/or COPD.

### Ethical considerations

The Dutch Law on Medical Research was not applicable to this study, according to the Medical Ethical Committee of the University Medical Centre Groningen (dossier: M21.286292), basically because no demanding or experimental intervention is involved. Written informed consent was obtained from patients and HCPs. Patient and HCP data were pseudonymized before being analysed. All data will be stored for 15 years and are available upon reasonable request from the authors. All study sites received financial compensation for participation.

### Reporting summary

Further information on research design is available in the Nature Research Reporting Summary linked to this article.

## Results

### Study sites and setting

Of the eight study sites, half were secondary care organisations (pulmonary outpatient departments of [university] hospitals), three were primary care organisations (general practices) and one was a nursing home and a specialised rehabilitation centre combined. In six study sites, one HCP participated in the study and in the two other study sites, two HCPs were involved. Of the participating HCPs, 10 were nurses specialised in lung diseases—general practice nurses or pulmonary nurses—and one HCP was a pulmonologist. Patient care concerned only consultations but varied from standardised monitor visits and consultations requested by the patient (poor symptom control) to consultations because of a referral from another HCP (diagnosis check, poor symptom control).

### Patient characteristics

Patient characteristics are provided in Table 1. Almost all study sites were able to include 10 patients, except for one study site that included 9 patients due to a job change by the HCP. The average age of the 79 participating patients was 60.5 (SD 16.3) and the majority was female (62%). Most patients lived together with, e.g. a partner (64.6%) and had (medical) social support available (57.1%).

**Table 1:** Patient characteristics in % of total or mean (SD) for the total patient population and the subgroups of asthma patients and COPD ± asthma patients (*N* = 79).

Characteristic	Instrument	Mean (SD) or % of total		
		Total	Asthma	COPD ± asthma
		(*N* = 79^a^)	(*N* = 37)	(*N* = 41)
Age		60.54 (16.27)	51.73 (18.65)	68.00 (7.96)
*Sex*				
Female		62.0%	70.3%	53.7%
*Educational level*				
Lower education		31.6% ^1^	19.4% ^2^	43.9%
Secondary education		38.0%	36.1%	39.0%
Higher education		29.1%	44.4%	17.1%
*Household composition*				
Living together		64.6% ^3^	74.3% ^4^	61.0%
Living alone		30.4%	25.7%	34.1%
Living in an institution		2.5%	0.0%	4.9%
*Social (medical) support available*				
*Yes*		57.1% ^5^	48.6% ^6^	63.4%
*Social (medical) support* ^b^				
Partner		45.6%	43.2%	48.8%
Child		7.6%	5.4%	9.8%
Parent		1.3%	2.7%	0.0%
Family member		2.5%	2.7%	2.4%
Friend/acquaintance		1.3%	0.0%	2.4%
Professional caregiver		7.6%	2.7%	9.8%
Other		3.8%	2.7%	4.9%
*Diagnosis*				
Asthma		46.0%	100%	0.0%
COPD		38.0%	0.0%	73.2%
Asthma and COPD		13.9%	0.0%	26.8%
Other^a^		1.3%	0.0%	0.0%
*% Medication non-adherence*				
TAI-12 < 54	TAI-12	81.0% ^7^	85.7% ^8^	76.5% ^9^
*Level of medication adherence*	TAI-12	45.8 (9.00) ^10^	44.2 (10.2) ^11^	47.3 (7.9) ^12^
*Illness control*	ACQ/CCQ		1.38 (0.89)	2.21 (1.16)
*Lung function*	FEV_1_%	67.5 (26.3) ^13^	79.6 (25.1) ^14^	54.9 (21.5) ^15^
*Exacerbations in the last year*				
Yes		38% ^16^	32.4% ^17^	43.9%
*Exacerbations per year*		1.7 (1.7)	1.8 (1.7)	1.7 (1.7)
*Comorbidity*				
Yes		62%	51.4%	70.7%
*Number of comorbidities*				
One		37.3% ^18^	47.6% ^19^	31.0% ^20^
Two		17.6%	9.5%	24.1%
>Two		45%	42.9%	44.8%
*Type of comorbidity*^b^				
Cardiovascular disease		40.5%	27.0%	51.2%
Diabetes mellitus		12.7%	10.8%	14.6%
Osteoporosis		6.3%	2.7%	7.3%
Depression		8.9%	5.4%	12.2%
Other		41.8%	37.8%	43.9%
*Number of medications*		5.3 (4.6) ^21^	3.9 (3.1) ^22^	6.3 (4.9) ^23^
*Type of inhalation medication*^b^				
Aerosol		73.4%	75.7%	70.7%
Dry powder inhaler		39.2%	37.8%	41.5%
Soft mist inhaler		11.4%	0.0%	22.0%

^1^*N*_missing_ = 1, ^2^*N*_missing_ = 1, ^3^*N*_missing_ = 2, ^4^*N*_missing_ = 2, ^5^*N*_missing_ = 2, ^6^*N*_missing_ = 2, ^7^*N*_missing_ = 16, ^8^*N*_missing_ = 9, ^9^*N*_missing_ = 7, ^10^*N*_missing_ = 16, ^11^*N*_missing_ = 9, ^12^*N*_missing_ = 7, ^13^*N*_missing_ = 14, ^14^*N*_missing_ = 4, ^15^*N*_missing_ = 9, ^16^*N*_missing_ = 2, ^17^*N*_missing_ = 2, ^18^*N*_missing_ = 28, ^19^*N*_missing_ = 16, ^20^*N*_missing_ = 12b, ^21^*N*_missing_ = 3, ^22^*N*_missing_ = 2, ^23^*N*_missing_ = 1

^a^Note that one patient used inhaled medication but had an unknown diagnosis.

^b^Total is not 100% since more than one option could be chosen.

In total 46% were diagnosed with asthma, 38.0% with COPD, 13.9% with asthma and COPD and 1.3% was diagnosed with another pulmonary disease. Patients with asthma scored an average of 1.38 (SD 0.89) on the ACQ, suggesting suboptimal controlled asthma. Patients with COPD scored an average of 2.21 (SD 1.16) on the CCQ, also suggesting suboptimal controlled COPD.

### Feasibility and implementation

Regarding potential reach, HCPs estimated to see an average of 28.5 (range 5–90) patients with COPD and/or asthma on a weekly base and predicted 74% (range 9–88%) of these patients to be non-adherent. On an annual basis, this would mean one HCP alone would see between 129 and 2314 patients (based on 180 workable days a year), and HCPs estimate 95–1712 patients would be non-adherent. Within this estimation, it is taken into account that patients could have multiple visits a year. The participating hospitals are estimated to see more patients a week compared to the primary healthcare organisations. Furthermore, the estimation of the percentage of non-adherence varied greatly amongst the HCPs which was independent of working in a primary or a secondary healthcare organisation.

In terms of usability, the mean SUS-score was 85.9. HCPs gave the highest scores (strongly agree) on the statements ‘I thought the TAI Toolkit was easy to use’, ‘I found the various functions in the TAI Toolkit were well integrated’ and ‘I would imagine that most people would learn to use the TAI Toolkit very quickly’. The statement ‘I think that I would like to use the TAI Toolkit frequently’ was scored relatively lower compared to the other positive statements.

Regarding broader aspects of acceptance, HCPs evaluated the TAI Toolkit to be ‘visual attractive’, with a ‘expedient lay-out and structure’ and ‘the instruction and information to be easily understandable and complete’. Additionally, HCPs stated the toolkit could be used as a ‘conversation starter’ or ‘communication medium’ and, with this, avoid tunnel vision towards the conversation and the patients. Using the TAI Toolkit during consultations could give a more comprehensive overview of- and insight into- the patient and the patient’s relation to his or her medication use since the medication use is questioned in a more structured and detailed way. HCPs suggested the TAI-questionnaire, in particular, gave more insight for the patient into his/her own behaviour.

Suggestions for improvement were given. Firstly, the TAI Toolkit is based on the TAI questionnaire and the patient is not always a reliable source. As one of the HCPs stated “patients are likely to give socially desirable answers”. With an unreliable outcome of the TAI questionnaire, HCPs can also not come to the most optimal intervention. Secondly, much of the information in the TAI Toolkit is known to lung-specialised HCPs. This makes the TAI Toolkit less attractive for continued use and may explain the lower score on the SUS-item ‘I think that I would like to use the TAI Toolkit frequently’ compared to the other SUS-items. HCPs explained the TAI Toolkit would be most useful to newly trained lung-specialists, non-specialists and as a reminder or as support with complex patient cases.

Some other considerations were to add some missing information like links to useful websites, to give more guidance within the toolkit when the referral was needed for the execution of an intervention, and to both digitalise the toolkit and reduce the size of the physical toolkit.

To enhance the implementation of the toolkit in daily practice, HCPs suggested a pocket-size or summarised model and/or a digital version, ideally integrated into the electronic patient record system. Regarding who could use the TAI Toolkit best, opinions were mixed. The majority of the HCPs suggested nurses (general and specialised) be the most appropriate considering their relation to the patient, amount of time available and more holistic view of the patient. However, some HCPs stated the TAI Toolkit could be used by any HCP because of the simplicity of the toolkit.

The majority stated that the interpretation of the TAI questionnaire and execution of most interventions demand specific knowledge and training (e.g., communication training for motivational interviewing, knowledge of physiopathology of lung diseases and on side-effects of respiratory medication). The interventions ‘link to daily habits’, ‘use daily reminders’ and ‘caregiver support’ were considered to be general HCP abilities, whereas the other interventions would require more specific knowledge. Additionally, suggestions were made for more guidance within the toolkit for referring when the HCP could not perform the intervention.

When it comes to location, all HCPs thought the toolkit could best be used at the general practice and outpatient clinics mainly because of time and expertise availability. Some suggested more locations, such as inpatients admitted to hospital for exacerbations of asthma or COPD, rehabilitation centres, nursing homes and community pharmacies with personnel trained appropriately.

When it comes to training, an introduction was desired, although the instruction within the toolkit itself was deemed sufficient. Most HCPs suggested a clinical lesson, but an instruction video was also proposed. Additionally, one HCP indicated the possible need for follow-up and evaluation after the introduction training. For promotion, HCPs suggested integrating the toolkit within existing courses and educational programs and dissemination through journals, congresses and informally via colleagues.

To stimulate maintained usage, the TAI toolkit could best be integrated within national protocols, guidelines and pre-existing educational programs. According to the HCPs, the TAI questionnaire and TAI Toolkit would ideally be integrated into electronic patient management systems, with pop-ups guiding their use in patients with asthma/COPD.

Updates and amendments of the TAI Toolkit were mostly preferred via a newsletter and when digital, automatically updated. On the other hand, HCPs pointed out the large volume of daily e-mails they receive. A webpage specifically for the TAI Toolkit with also information on updates was suggested as alternative.

### Medication adherence and interventions

Most common reasons for suspecting non-adherence by HCPs were (1) persistence or aggravation of symptoms, (2) non-adherence was already established (e.g. using electronic prescription/dispensing review, during regular consultations, admission, new patient) or (3) the patient told she/he did not use the medication as agreed upon. In six patients, no particular reason for suspecting non-adherence and applying the toolkit was noted.

The mean score on the TAI-12 was 45.8 (SD 9.0), with 81% of the included patients being considered medication non-adherent (i.e., TAI < 54). Similar results were found in patients with asthma as in patients with COPD (±asthma), although patients with COPD were slightly more adherent (Table 1).

**Fig. 3:**
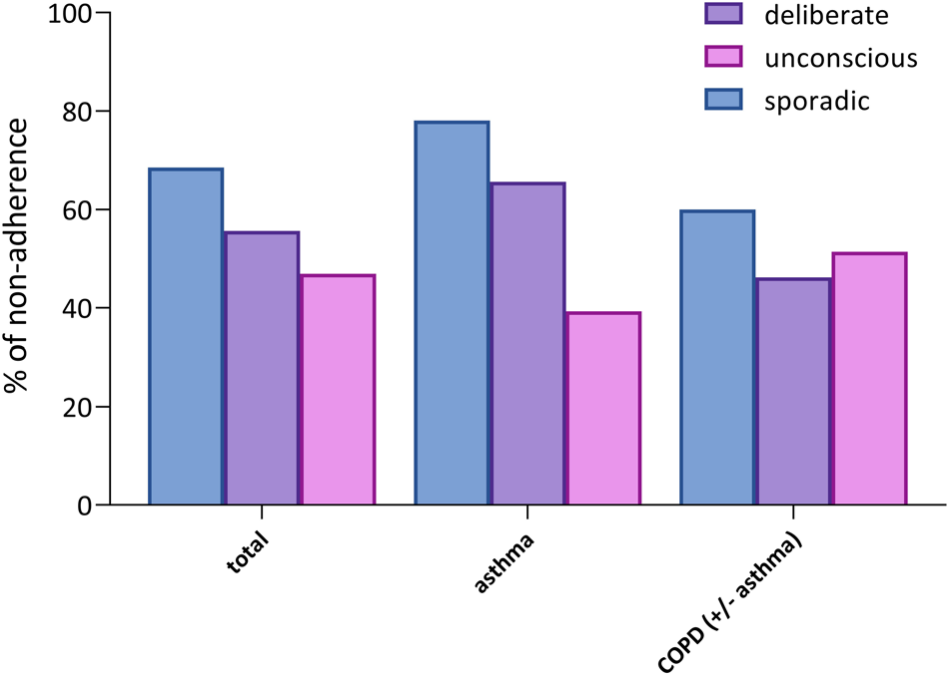
Non-adherence in the total study population (N = 79) and the subgroups asthma (N = 37) and COPD ± asthma (N = 41).

The most common type of non-adherence was sporadic non-adherence (69%, score<25 on items 1–5). The least common type was unconscious non-adherence (47%, score<4 on items 11–12) (Fig. 3). Furthermore, patients who were sporadically non-adherent were also more likely to be deliberate non-adherent (*P* < 0.001). No correlation between unconscious non-adherence and the other two types of non-adherence was found. Note that we defined non-adherence as not fully adherent by not scoring a maximum score on the TAI or subscores of the TAI, and patients could be simultaneously sporadic, deliberate or unconscious non-adherent.

**Fig. 4:**
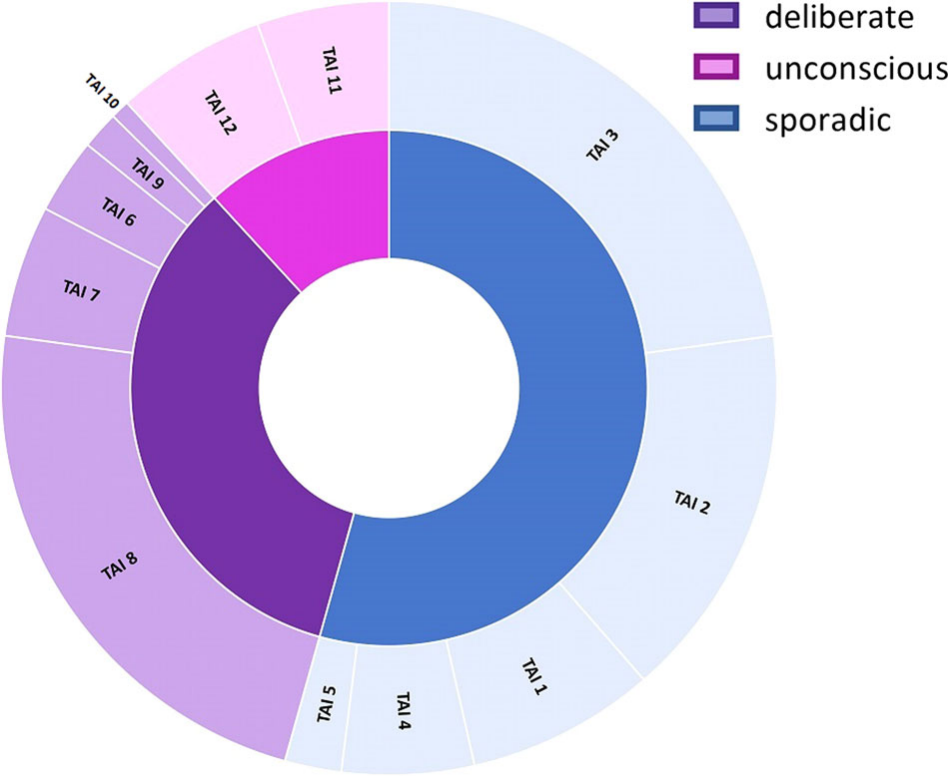
Frequency of non-adherence reasons reported by patients depicted by TAI item (outer circle) and type of nonadherence (inner circle).

The TAI-items that were generally scored the lowest were TAI-item 3 “stop taking inhalers when feeling well”, TAI-item 8 “taking fewer inhalers than prescribed” and TAI-item 2 “forget your inhalers” (Fig. 4).

**Fig. 5:**
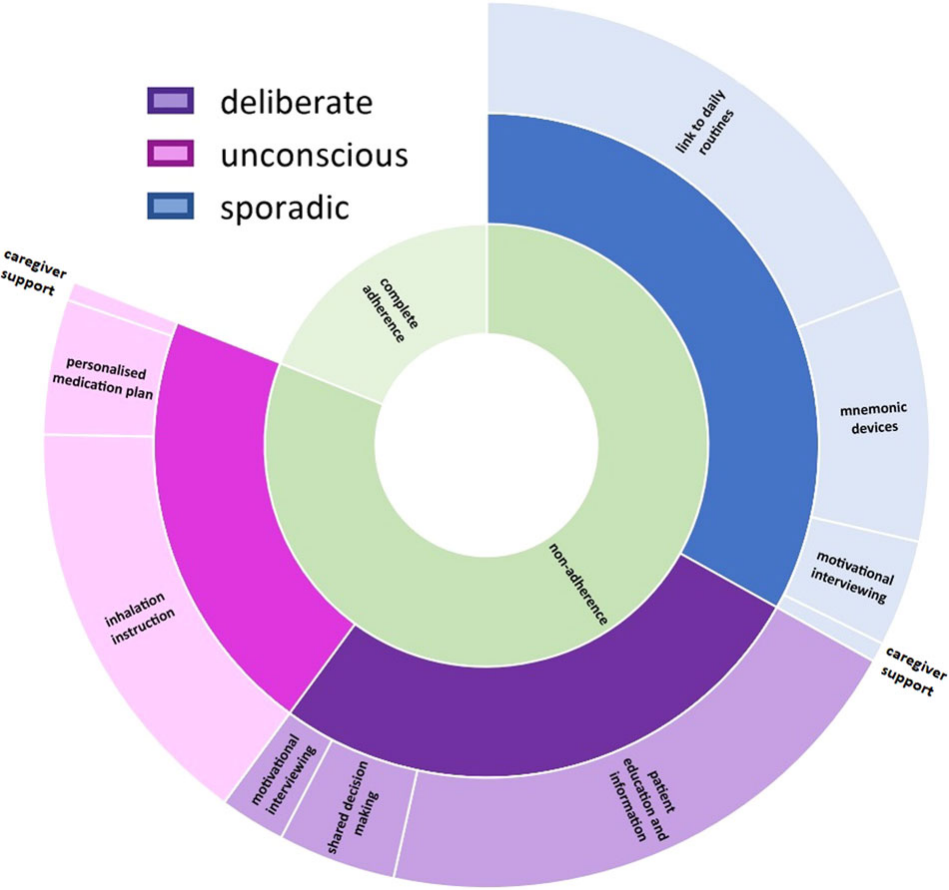
Number of times the interventions have been selected within the type of non-adherence (inner circle: adherence vs non-adherence, middle circle: type of non-adherence, outer circle: intervention selected).

Interventions suggested by the TAI Toolkit, which were most often selected, were *patient education and information, link to daily routine* and *inhalation instruction* (Fig. 5).

## Discussion

Overall, the TAI Toolkit showed good usability and potential for larger-scale implementation. Suspicions of non-adherence by HCPs were confirmed by the TAI in over 80% of the patients. Of note, patients varied in terms of demographic and clinical characteristics. Sporadic non-adherence was the most common type of non-adherence identified.

The TAI Toolkit is the first tool of its kind and shows good usability. The need for easy-to-use measurement methods to detect non-adherence and guidance on how to act on the outcome of those measurements has been stressed before^10^. Indeed, a systematic review identified the TAI questionnaire as one of the best self-report adherence instruments in terms of psychometric quality, accessibility and utility for detecting non-adherence in patients with asthma^10^. The review also disclosed the TAI Toolkit as the only instrument that has been further developed and used to guide HCPs into the deployment of cause-based and patient-tailored interventions in patients with lung diseases^10^. All interventions recommended by the TAI Toolkit are evidence-based. The added value of the TAI Toolkit is that it identifies the specific type of non-adherence (sporadic, deliberate, unconscious) and then offers a multifaced tailored intervention. Multifaced tailored interventions can have a significant impact on improving medication adherence compared to usual care in asthma/COPD and in other chronic patient groups, demonstrating the potential impact of the TAI Toolkit for patients using inhalation medication^11–15^. The potential impact in terms of the reach of the TAI Toolkit is also presented within this study. The estimation of encounters with relevant patients is measured subjectively and varied between the participating study sites. However, the overall high numbers of relevant patients HCPs encounter give an impression of and nuance to the experienced relevance and usefulness of the toolkit. Also, it gives insight into the relevance of the participating study sites and, therefore, the potential settings.

Regarding patient characteristics with suspicion of non-adherence, patients were not only heterogeneous in terms of physical status but also with respect to age, comorbidities and socioeconomic background. However, the overall older population, with multimorbidity and polypharmacy included in this study, is known to be at higher risk for nonadherence^16–18^. Patients’ number of exacerbations was as expected for the general population of both asthma and COPD, but lower than expected looking at the high levels of non-adherence we found in this study^19–22^.

Notably, whereas non-adherence in patients with asthma and COPD is estimated to be between 22% and 78%, we found a higher percentage of 81% for complete non-adherence and an even higher percentage in patients with asthma (85.7%)^3^. This relatively high degree of non-adherence could be explained by the fact that we defined “non-adherence” as not completely adherent (i.e. TAI < 54) in line with other studies using the TAI questionnaire^23,24^. Another explanation for this high degree of non-adherence is that one of the inclusion criteria was to select patients with a suspicion of non-adherence. However, note that previous studies indicated that HCPs frequently overestimate adherence in their patients^25,26^.

We found sporadic non-adherence to be the most common type of non-adherence with forgetfulness and misunderstanding of the therapy the most common in both patient groups. In line with previous studies, unconscious non-adherence was more common in patients with COPD, and deliberate non-adherence was more common in patients with asthma^23,24^. Patients with COPD are often lower educated and of older age compared to patients with asthma (possibly leading to more misunderstanding and forgetfulness), whereas patients with asthma are more likely to be deliberately non-adherent due to more fluctuating asthma symptoms^23,27,28^. Here, it is important to note that although some non-adherence barriers are patient reported, this does not mean the patient is accountable. For example, inadequate patient knowledge regarding when or how to use the inhaler can also be the result of poor HCP education or communication.

Not surprisingly, when considering the most common causes of non-adherence, the interventions ‘patient education and information’ and ‘link to daily routine’ were the most selected interventions. Also, inhalation instruction was one of the most selected interventions, while inhalation technique failure was not marked as one of the most common causes of non-adherence. That said, even though the TAI Toolkit offers an overview of the causes of non-adherence coupled interventions, HCPs—and the patient—still have the task of detecting what is the most prominent or underlying cause. For instance, when a patient believes medication is not that effective or necessary (conscious non-adherence), he or she may also sooner forget to take the medication (sporadic non-adherence).

Finally, inhalation instruction and patient education are often perceived as usual care when starting or switching inhalation medication, but selecting these interventions when non-adherence was observed could suggest that reinforcement was required.

This study has multiple strengths. First, the TAI Toolkit has been designed with a variety of team members (researchers, nurses, pharmacists and physicians), making the toolkit accessible for different HCPs. Also, the interventions advised by the toolkit have been proven to enhance medication adherence and some have already been implemented in current guidelines^29,30^, which meant that the interventions could be easily and instantly delivered by the HCPs. Third, the prototype has been tested in a variety of settings (rehabilitation centre/nursing home, hospital and general practice), adding to the generalisability of the results.

There are also some limitations. First, although the prototype has been tested in different settings, all settings were outpatient consultation rooms in a high-income country. Different settings, such as nursing wards and community pharmacies, may be relevant settings as well. Second, the participating HCPs were all specialised to some degree in respiratory medicine and patients were only included during consultations concerning their asthma/COPD. It remains uncertain whether general nurses, pharmacists and physicians (not-specialised in lung care) would be able to use the TAI Toolkit and what the most relevant or crucial moments of encounters are to apply the toolkit.

Third, one of the inclusion criteria was a suspicion of non-adherence. This leads to targeting the right population but possibly also to inflation of the observed percentage of patients that were non-adherent within this study. Also, suspicion of non-adherence is a rather subjective inclusion criterion and depends greatly on the HCP. This inclusion criterion could have led to a selection bias, e.g., patients who felt more connected with their HCP would perhaps feel more comfortable discussing barriers or difficulties with their medication compared to patients who felt less connected and the HCP would sooner find clues for non-adherent behaviour.

Lastly and most importantly, the TAI questionnaire and, therefore, the toolkit do give insight into patients' reported behaviour concerning medication, but there are more underlying factors that play a role. Factors (or causes) that contribute to non-adherence, such as prescription errors (or non-adherence to treatment guidelines by HCPs), availability of the medication and the possibility that the medication simply does not result in the desired treatment effect, are not directly identified by the TAI. When using the toolkit, it is the HCP’s task to not just view the answers and scoring; they are expected to discuss deviants (or lower scorings). Even though our results show that HCPs experience more insight into patients’ behaviour, the effect of a potential intervention is highly dependent on the communication skills of the HCP.

More research is needed that focuses on the different types and causes of non-adherence in lung patients. Moreover, more research is needed to identify the crucial moments, HCPs’ abilities to identify the most prominent cause of non-adherence and if, consequently, the appropriate intervention is applied and effective. With non-adherence also being mentioned generally and non-specifically in the Dutch guidelines for asthma and COPD, if and how non-adherence has been detected and managed currently is unclear^31,32^. Furthermore, the impact of an intervention does not only depend on whether the (most prominent) cause of non-adherence is targeted but also on the patient characteristics and quality of the application of the intervention itself. Insight into the relevance of interventions for specific subpopulations is desired as well.

For use in clinical practice and the possibility for upscaling, the TAI Toolkit needs to be improved and further developed according to the feedback retrieved in this study; a smaller pocket-size model and a digital version—preferably integrated into electronic patient record software—were desired. In addition, minor adjustments to the content were suggested, such as an overview of all possible involved healthcare professionals and organisations. Because of the longer consultation time of nurses and the more holistic view of the patient within the nursing profession, as mentioned by the HCPs, further development and implementation should focus on the nursing profession. Additionally, the TAI Toolkit should be tested and evaluated in other contexts, e.g. in nursing wards, in other healthcare disciplines and by non-lung specialists to assess the potential for wider implementation.

Secondly, patient-reported measurements such as the TAI are always prone to self-report biases and although the TAI also provides the causes for non-adherence, it is advisable to complement with another objective measurement method such as smart inhaler data or medication refill rates^33^.

Lastly, evaluating whether and why a patient is non-adherent remains a challenge and the TAI questionnaire and toolkit do not cover all possible factors and causes. Deployment of the TAI questionnaire and the TAI Toolkit should be considered as a low-key routine tool, but HCPs must remain aware of less visible or straightforward causes for non-adherence, such as the possibility the prescribed medication simply does not have a beneficial effect on the patient. Understanding the patient and his or her health behaviour is key to guide patients into more adherent behaviour^33^. This requires strong communication skills and a relationship of trust between the HCP and the patient. A focus on HCPs’ communication skills and training is needed to tackle the issue of non-adherence and to be able to use the TAI Toolkit properly.

The TAI Toolkit is the first in its kind to combine and connect an adherence measurement instrument to proven effective interventions. The toolkit was deemed feasible for daily practices specialised in chronic lung care and usable for HCPs to structurally acquire more understanding of non-adherent behaviour. Although the effectiveness of the TAI Toolkit was beyond the scope of this research, the TAI Toolkit has the potential to improve suboptimal adherence and clinical outcomes starting by structurally detecting and addressing non-adherence and its causes.

### Supplementary information

Supplementary Material

Reporting Summary

## Data Availability

The data that support the findings of this study are available from the corresponding author upon request. All data provided will be anonymised and terms for usage will be agreed in advance.
