# Supplementary material for: Usability and feasibility of the Test of Adherence to Inhalers (TAI) Toolkit in daily clinical practice: The BANANA study
Source: NPJ Prim Care Respir Med. 2024 May 28;34:13. doi: 10.1038/s41533-024-00372-z (PMC11133447; doi:10.1038/s41533-024-00372-z)
Supplement: Supplementary file 1 — Supplementary Material [file 41533_2024_372_MOESM1_ESM.pdf]

Supplementary materials

Achterbosch et al. Usability and feasibility of the Test of Adherence to Inhalers (TAI) Toolkit in daily clinical practice: The BANANA study. *npj Prim Care Respir Med.*

Appendix 1 – case report form

This case report form consists of four parts and need to be completed after you applied the TAI Toolkit to the patient. You can with use of the checkboxes below keep up with parts you have already completed

☐ Part I consists questions concerning asthma or COPD control

☐ Part II consists socioeconomic questions

☐ Part III consists questions concerning health

☐ Part IV consists questions concerning the TAI

Attention!	Part I and II are to be filled in together with the patient since not all this information can be retrieved from the EPD. Part III and IV can be filled in with information from the EPD.
Attention!	Note on every page the date and case number from the attached code list and note on the code list the corresponding patient number of name of the patient.
Attention!	Save and send the TAI with the corresponding case report form. Staple the TAI and case report form together.

I Questions concerning asthma or COPD control

1. ACQ/CCQ

* Instruction: discuss with the patient

2. a. Has the patient had exacerbations over the last year? *Yes (continue to b)/No (continue to part II)*
   b. How many times has the patient had exacerbation in the last year. Fill in the number of applicable treatments. *Number of exacerbations ... treatment with 1) short-acting bronchodilators ...; 2) other medication (e.g. prednisone or antibiotics) ...; 3) visit to ER was necessary...; 4) hospitalisation was necessary ...; 5) a combination of the above mentioned treatments ...*

II Socioeconomic questions

*Instruction: discuss with patient or let patient fill in this part of the form.

1. What is your educational level? *a. lower education (primary education, vmbo, first three years of havo/vwo, entrance education, mbo1); b. secondary education (last two or three years of havo/vwo, mbo2-mbo4); c. higher education (university, applied science bachelor or master)*
2. What is the composition of your household? *a. living together (with partner, family member, roommate, parents, children, different); b. living alone; c. living in an institution (e.g. elderly home or asylum centre).*
3. *a.* Is there someone in your environment who could help you with your medication e.g. help remembering medication intake or come along to your physician or pharmacist? *Yes (continue to 3b)/ No (continue to section III)*
   b. Who in your environment could help you with your medication, e.g. help remembering the medication intake or come along to your physician or pharmacist? a. partner; b. child; c. parent; d; family member; e. friend; f. professional caretaker; g. other, namely …

III Question concerning health

*Instruction: collect information from EPD

1. Age …
2. Sex *female/male/other*
3. Indication for inhalation medication *asthma/COPD/asthma and COPD, other namely...*
4. a. Is there comorbidity? *Yes, there are one or more diagnoses besides asthma/COPD (continue to question 4b)/ No (continue to question 5)*
   b. How many diagnoses are made beside the asthma/COPD? *one/two/more than two*
   c. What are the comorbidities? *cardiovascular/diabetes mellitus/osteoporosis/depression/other, namely ...*
5. a. How many medication does the patient use beside the inhalation medication? This also includes medical ointments, powders, medicinal drinks …
   b. What kind of inhalation medication is used? *earosol/powder inhalation/ oft mist inhalation*
6. What where the last spirometry data and from which date? *a. unknown; b. date ...(year) ... (month); FEV1, FVC, FEF25-75, PEF, Tiffeneau (%pred and z-score)*

IV Questions concerning the TAI

*Instruction: please fill in after applying the TAI Toolkit

1. What was the cause for suspicion of non-adherence?
2. What was the total score on the TAI?
3. What where the TAI-items with the lowest scores? Select max. 3 items.
4. Which interventions from the TAI Toolkit did you select? Select max 3 interventions.

END OF THE CASE REPORT FORM

Appendix 2 - interview

1. [date]
2. [name of health care professional]
3. You are works as [a. pulmonologist, b. general practitioner, c. nurse, d. pulmonary nurse, e. general practice nurse, f. physician assistant, g. other, namely …]
4. You work in [a. primary care, b. secondary care, c. tertiary care]
5. You work at [a. University Medical Centre Groningen, b. Martini Hospital Groningen, c. Ikazia Hospital Rotterdam, d. Radboud University Medical Centre Nijmegen, e. Groenhuysen, f. Health Practice Sûnhûs Makkum, g. General Practice Wateringse Veld Den Haag, h. University Medical Centre Heyendael Nijmegen]
6. Are you specialized in health care for people with COPD and asthma? Here, 'specialized' means that you have followed additional training or education in health care for lung patients [a. no, b. yes, give an clarification …]
7. Give a brief description of your work activities and how your daily activities are related to patients with COPD and asthma.

8. How many patients with asthma and COPD do you encounter during you daily work activities on average?
9. In how many patients do you estimate medication non-adherence? Give an average per week.
10. How many patients did you on ask to participate in this research on estimate?
11. How many patients were willing to participate in this research?
12. What where the barriers for patients to not participate in this research?

13. System Usability Score (score from 1 (strongly disagree) to 5 (strongly agree)
    1. I think that I would like to use the TAI Toolkit frequently.
    2. I found the TAI Toolkit unnecessarily complex
    3. I thought the TAI Toolkit was easy to use.
    4. I think that I would need the support of a technical person to be able to use the TAI Toolkit.
    5. I found the various functions in the TAI Toolkit were well integrated.*
    6. I thought there was too much inconsistency in the Toolkit.
    7. I would imagine that most people would learn to use the TAI Toolkit very quickly.
    8. I found the TAI Toolkit very cumbersome to use.
    9. I felt very confident using the TAI Toolkit.
    10. I needed to learn a lot of things before I could get going with the TAI Toolkit.
    *Here, with integrated it is meant if the TAI questionnaire and TAI Toolkit are well adapted to each other.

14. Name three positive features of the TAI Toolkit and explain. These features can be related to the content – e.g. the information and instruction manual– as is can be related to the design – e.g. is the current design appropriate for your work activities.
15. Name three point of improvement of the TAI Toolkit and explain. These features can be related to the content – e.g. the information and instruction manual– as is can be related to the design – e.g. is the current design appropriate for your work activities.
16. The TAI Toolkit is currently designed as a physical folder with tabs. In what form would the TAI Toolkit be best suited for your work activities? Please, give an explanation. [a. physical (on paper), b. digital, c. different, namely …]

17. During what work activities or where in health care could the TAI Toolkit best be put into practice? Please, give an explanation. [a. during regular consultation at e.g. the GP,

pulmonologist or GP nurse, b. during hospitalization, c. during admission at the emergency department, d. at the pharmacy, e. other, namely …]

18. Which health care professional is most fitted to use the TAI Toolkit (consider the available time and professional focus). Select all applicable. Please, give an explanation. [a. pulmonologist, b. general practitioner, c. nurse, d. pulmonary nurse, e. general practice nurse, f. physician assistant, g. other, namely …]

19. The execution of the interventions recommended in the TAI Toolkit was not a part of this study. If you were to execute the recommended interventions, how much time (in minutes, hours, days or weeks) would it take to execute? Please, give an estimation for the three interventions you selected the most.
20. The execution of the interventions recommended in the TAI Toolkit was not part of this study. If you were to execute the recommended interventions, would you have been capable to execute the intervention yourself? Please, answer with yes or no for the interventions you selected in the previous question. And if selected no, give an explanation.

21. When the TAI Toolkit is to be used for the first time, do you need training or instruction? Please, give an explanation.
22. For the execution of the recommend intervention in the TAI Toolkit, do you need training or instruction?
23. For which interventions do you need training or instruction to be able to execute the intervention? Select all appliable. Please, give an explanation.
24. What are the best means and methods to promote the TAI Toolkit within and outside your organisation? Consider means and methods such as newsletters, clinical lessons, publication in professional (scientific) magazine. Please, give an explanation.
25. How can the TAI Toolkit be implemented within your organisation? Consider procedures such as securing the use of the TAI Toolkit within a protocol or guideline from your organisation. Please, give an explanation.
26. In case of changes or updates of the TAI Toolkit, how can the users of the TAI Toolkit be updated of these changes and updates? Consider means and methods such as newsletters or a website. Please, give an explanation.
